# Integrated genomic and immunophenotypic profiling reveals monoclonal origin, smoking-driven evolution and heterogeneous microenvironment in pulmonary adenosquamous carcinoma

**DOI:** 10.3389/fimmu.2026.1865105

**Published:** 2026-06-15

**Authors:** Xiuwen Zhang, Heng Wu, Wenhao Zhao, Zixuan Hu, Guannan Wang, Yongwen Li, Xuanguang Li, Hongbing Zhang, Minghui Liu, Chen Chen, Jinghao Liu, Wenhao Zhou, Hongyu Liu, Jun Chen

**Affiliations:** 1 Department of Lung Cancer Surgery, Center of Thoracic Surgery, Tianjin Medical University General Hospital, Tianjin, China; 2 Department of Thoracic Surgery, Nantong First People’s Hospital, Nantong, Jiangsu, China; 3 Tianjin Key Laboratory of Lung Cancer Metastasis and Tumor Microenvironment, Tianjin Lung Cancer Institute, Tianjin Medical University General Hospital, Tianjin, China; 4 Kindstar Global Precision Medicine Institute, Wuhan, Hubei, China

**Keywords:** evolutionary trajectory, multiplex immunofluorescence, pulmonary adenosquamous carcinoma, tumor microenvironment, whole-exome sequencing

## Abstract

**Background:**

Pulmonary adenosquamous carcinoma (ASC) is a rare, aggressive subtype of non-small cell lung cancer (NSCLC), characterized by both adenocarcinoma (ACC) and squamous cell carcinoma (SCCC) components. Due to its rarity, the clonal origin, evolutionary drivers, and tumor microenvironment (TME) of ASC remain poorly understood.

**Methods:**

We conducted clinical and prognostic analyses on 76 ASC patients. Nine patients with available tissues underwent microdissection to isolate matched ACC and SCCC regions, which were then analyzed using whole-exome sequencing (WES), copy-number analysis, phylogenetic reconstruction, and multiplex immunofluorescence (mIF) for immune profiling.

**Results:**

Multivariate Cox regression identified the histological subtype of the ACC component as an independent prognostic factor, with solid and micropapillary subtypes associated with the poorest overall survival. WES revealed frequent mutations in TP53 (56%), EGFR (33%), MET (33%), and PIK3CA (22%) across tumor regions. Shared truncal mutations and copy-number alterations between ACC and SCCC regions suggest a common monoclonal origin. Smokers had a significantly higher tumor mutational and neoantigen load than non-smokers, with greater overlap in mutations between ACC and SCCC. Phylogenetic analysis showed distinct evolutionary patterns: smokers’ tumors displayed smoking-related and APOBEC mutational signatures, while non-smokers had aging-related signatures. mIF analysis revealed significantly higher immune cell infiltration (CD8^+^ T cells, CD4^+^ T cells, B cells, macrophages, CAFs) in ACC regions compared to SCCC.

**Conclusion:**

Our findings support a monoclonal origin for ASC, with smoking influencing divergent evolutionary trajectories and immune microenvironment characteristics between ACC and SCCC. These insights provide a molecular framework for personalized ASC therapies.

## Introduction

Lung cancer has the highest incidence and mortality rates worldwide, accounting for approximately 12.4% of all newly diagnosed malignancies (1). Histologically, the disease is classified into small cell lung cancer (SCLC) and non-small cell lung cancer (NSCLC), each with distinct morphological and clinical characteristics. NSCLC constitutes 80–85% of all lung cancer cases and includes lung adenocarcinoma (LUAD; ~50%), lung squamous cell carcinoma (LUSC; ~20–30%), and, less commonly, large cell carcinoma and other subtypes (2–4). LUAD predominantly occurs in females and non-smokers and is characterized by canonical driver alterations including EGFR or KRAS mutations and EML4-ALK translocations, whereas LUSC primarily affects male smokers despite lacking consistently defined driver genes (5, 6). Targeted therapies have demonstrated substantial efficacy in LUAD by specifically inhibiting known driver mutations, such as EGFR and ALK; however, comparable success has yet to be achieved for LUSC (7, 8).

Pulmonary adenosquamous carcinoma (ASC) is a rare and aggressive histological subtype of non-small cell lung cancer (NSCLC), accounting for approximately 0.4–4% of all lung cancers (9). According to the World Health Organization (WHO) classification, ASC is defined by consisting both adenocarcinoma components (ACCs) and squamous cell carcinoma components (SCCCs), each comprising at least 10% of the tumor (10). Notably, ASC exhibits marked morphological heterogeneity and is not merely a combination of both NSCLC subtypes — LUAD and LUSC — but rather, it represents a distinct entity with unique clinical features, with more aggressive behaviors and poorer prognosis outcomes when compared to pure LUAD or LUSC (11). Currently, no specific treatment guidelines are tailored to ASC; clinical management generally follows standard NSCLC protocols, with surgery and platinum-based chemotherapy as mainstays (12, 13). Although targeted therapies can be used as a first-line treatment for advanced ASC harboring *EGFR* mutations or *ALK* rearrangements, the overall therapeutic landscape remains limited, partly due to an incomplete understanding of its molecular underpinnings.

The cellular origin and clonal evolution of ASC remain subjects of active debate. Previous studies have primarily focused on oncogenic mutations and reported that ASC and LUAD have similar mutation spectrum, including *EGFR* alterations (14, 15). Next-generation sequencing studies have identified some shared mutations in between ACCs and SCCCs components within individual ASC tumors, potentially suggesting their derivation from a common ancestral cell. For instance, Krause et al. conducted whole-exome sequencing (WES) on three ASC cases and reported that ACCs and SCCCs harbored shared truncal mutations, along with distinct branch -specific driver mutations (12). These findings supported a common clonal origin and suggested the possible transformation of ACCs to SCCCs (12). However, comprehensive insights into the molecular drivers of this divergence and the subsequent evolutionary trajectories-particularly the influence of environmental factors such as smoking-particularly remain limited. Furthermore, the tumor microenvironment (TME) plays a crucial role in cancer progression and therapy response. While immune checkpoint blockade has revolutionized NSCLC treatment, the distinct immune landscapes of ACC and SCCC components within ASC, and their implications for immunotherapy, are virtually unexplored. Previous studies have shown that when compared to LUAD, LUSC more frequently expresses PD-L1 and shows greater immune cell infiltration, including CD8^+^ T cells and macrophages et al. (16). LUSC also harbors a higher somatic mutation frequency and displays greater immunogenicity, presenting enhanced therapeutic opportunities for patients with advanced disease. However, specific TME characteristics of ACCs and SCCCs in ASC remain poorly understood.

In this study, we performed an integrated clinical, genomic, and immunophenotypic analysis of ASC. We first retrospectively analyzed a cohort of 76 ASC patients and identified the ACC histological subtype as an independent prognostic factor of ASC. Subsequently, for nine representative cases, we employed microdissection to physically separate ACC and SCCC components, followed by WES to delineate somatic mutations, copy-number alterations, and clonal architecture. Phylogenetic reconstruction and mutational signature analysis were applied to infer evolutionary histories. Finally, we utilized multiplex immunofluorescence (mIF) to quantitatively compare the immune cell and stromal landscapes between the two histological components. This multi-faceted study aims to provide a comprehensive understanding of the biology of ASC, clarifying its clonal origin, elucidating its evolution, and characterizing its immune microenvironment, thereby informing future strategies for precision therapy.

## Materials and methods

### Patient enrollment and tissue collection

We retrospectively reviewed patients diagnosed with primary ASC who had complete medical records at Tianjin Medical University General Hospital. In total, 76 eligible ASC patients were enrolled. Patient diagnoses were independently confirmed by two board-certified pathologists who reviewed hematoxylin and eosin (H&E) and immunohistochemistry (IHC) slides. Follow-up was conducted via telephone, and survival time was defined as the interval from the date of surgery to either the date of the last follow-up or death. Formalin-fixed paraffin-embedded (FFPE) tumor samples from nine ASC patients with sufficient tissue quality were selected for genomic and multiplex immunofluorescence (mIF) analyses. Immunohistochemical markers, including TTF-1, Napsin A, P63, and P40, were used to distinguish between ACCs and SCCCs. Guided by H&E staining, manual microdissection was performed to separate ACC and SCCC regions for downstream genetic assays. For each case, 15–20 consecutive unstained tissue sections (5-μm thick) were mounted on glass slides. Based on prior H&E and IHC results, morphologically distinct tumor components and matched normal lung tissues were identified and marked. Target tissue was then carefully scraped from the slide using a sterile disposable surgical blade.

Patients provided written informed consent prior to enrollment. The study was approved by the Institutional Ethics Committee of Tianjin Medical University General Hospital(IRB2024-YX-045-01).

### WES and reads alignment

WES was performed by Yucebio Technology (Shenzhen, China). Genomic DNA was extracted using the Mag-Bind Blood & Tissue DNA HDQ 96 Kit (QIAGEN, Germany) and quantified using the dsDNA High Sensitivity Assay Kit (Thermo Fisher Scientific, Massachusetts, USA). Sequencing libraries were prepared using the Exome Plus Panel V.1.0 (IDT, Iowa, USA) and sequencing was conducted on the BGI-T7 platform, generating 100 bp paired-end reads. Adapter trimming and low-quality reads filtering were conducted using SOAPnuke. Clean reads were aligned to a human reference genome (hg19) using BWA (v.0.7.12), and duplicate reads were removed using Sambamba (v.0.5.4). Resulting BAM files were then used for downstream analyses.

### Somatic variant calling

Somatic mutations, including single nucleotide variants (SNVs) and small insertions and deletions (Indels), were identified by comparing sequencing data from tumor tissues and matched adjacent non-tumor tissues using VarScan (v.2.4). Variants with a low variant allele frequency (VAF < 0.02) or insufficient read depth (<10 reads in either tumor or normal samples) were excluded. The remaining mutations were annotated using SnpEff (v.4.3) with the National Center for Biotechnology Information RefSeq database (https://www.ncbi.nlm.nih.gov/refseq/). Tumor mutational burden (TMB) was defined as the number of non-synonymous somatic mutations/megabase (mut/Mb) of sequenced coding region. The tumor neoantigen burden (TNB) was similarly defined as the number of predicted neoantigens/megabase.

A non-synonymous mutation oncoplot was generated using the R package maftools (v.2.8.05), based on genes reported by Bailey et al. as significantly mutated in classical LUAD and LUSC according to The Cancer Genome Atlas (TCGA) dataset (17).

### Cancer cell fraction estimation and clonality analysis

PyClone (v.0.13.1) (18) was used to estimate the number of clones and calculate the CCF of inferred mutational clusters as described by Landau et al. (19). A mutation was classified as clonal if the CCF harboring it was >0.95 with probability >0.5 and subclonal otherwise (19).

### Copy number variation analysis

ASCAT-NGS (v3.1.0) was implemented for somatic CNV calling from WES alignments, with integrated correction for GC-content biases, tumor purity/ploidy normalization, and differential analysis against matched germline controls. Based on segmented copy number profiles generated by ASCAT-NGS, the GISTIC2.0 algorithm was used to identify significantly recurrent copy number alterations across the cohort. All analyses were conducted using log2-transformed data, with values constrained between −1 and 1 during data preprocessing (before input to GISTIC) to reduce over-segmentation artifacts. These clamping thresholds were particularly useful for minimizing noise in regions with extreme values caused by discrepancies in attenuation curves between adjacent probes.

### Phylogenetic analysis and mutational signatures

Maximum parsimony trees were constructed for each case using binary presence/absence matrices based on the repertoire of non-synonymous and synonymous somatic mutations, gene amplifications, and homozygous deletions in tumor samples, as described by Murugaesu et al. (20, 21). Mutational signature decomposition was performed using the R package deconstructSigs, referencing the 30 COSMIC mutational signatures (signature.cosmic) previously reported in LUAD and LUSC (22, 23).

### mIF

mIF staining and initial evaluations were performed by Yucebio Technology (Shenzhen, China). Briefly, 5-µm-thick sections were dewaxed from FFPE blocks in xylene and rehydrated through a graded ethanol series, followed by fixation in 10% neutral buffered formalin for 10 minutes. Slides were stained with the following primary antibodies: anti-CD4 (ab133616, Abcam, Cambridge, Britain), anti-CD8 (MA1-80231, Thermo Fisher Scientific, Massachusetts, USA), anti-CD20 (48750S, Cell Signaling Technology, Boston, USA), anti-CD68 (ZM-0060, Amresco, USA), anti-α-SMA (ab244177, Abcam, Cambridge, Britain), and anti-pan cytokeratin (ab234297, Abcam, Cambridge, Britain). After incubation with antibodies, slides were treated with a blocking buffer for 10 minutes, followed by a secondary horse radish peroxidase-conjugated polymer antibody and tyramide signal amplification. Nuclear staining was performed using 4’,6-diamidino-2-phenylindole (DAPI) for 10 minutes. Imaging was conducted using the Vectra Polaris system (Akoya Biosciences, Massachusetts, USA), and standard image analysis was performed using Halo Link software (Indica Labs, New Mexico, USA). Boxplots were generated using the ggplot2 R package (v.3.5.0). Immune and stromal cell populations were labeled using specific markers: CD8 for CD8^+^ T cells, CD4 for CD4^+^ T cells, CD20 for B cells, CD68 for macrophages, and α-SMA for CAFs. Tumor regions were identified by Pan-cytokeratin (Pan-CK) staining.

### Statistical analysis

The primary endpoint of this clinical study was overall survival (OS). Intergroup comparisons were performed using Student’s t-test, one-way ANOVA, or χ²-test (Chi-square test) as appropriate for variable types. Univariate survival analysis was conducted with the Kaplan-Meier method, and between-group differences were assessed by the log-rank test. Multivariate survival analysis utilized the Cox proportional hazards regression model, with the omnibus test evaluating the overall model significance. *P* < 0.05 was defined as the threshold for statistical significance. All statistical computations were executed using SPSS Statistics, Version 27.0.1 (IBM, New York, USA).

## Results

### Clinical characteristics and prognostic factors in ASC

**Figure 1:**
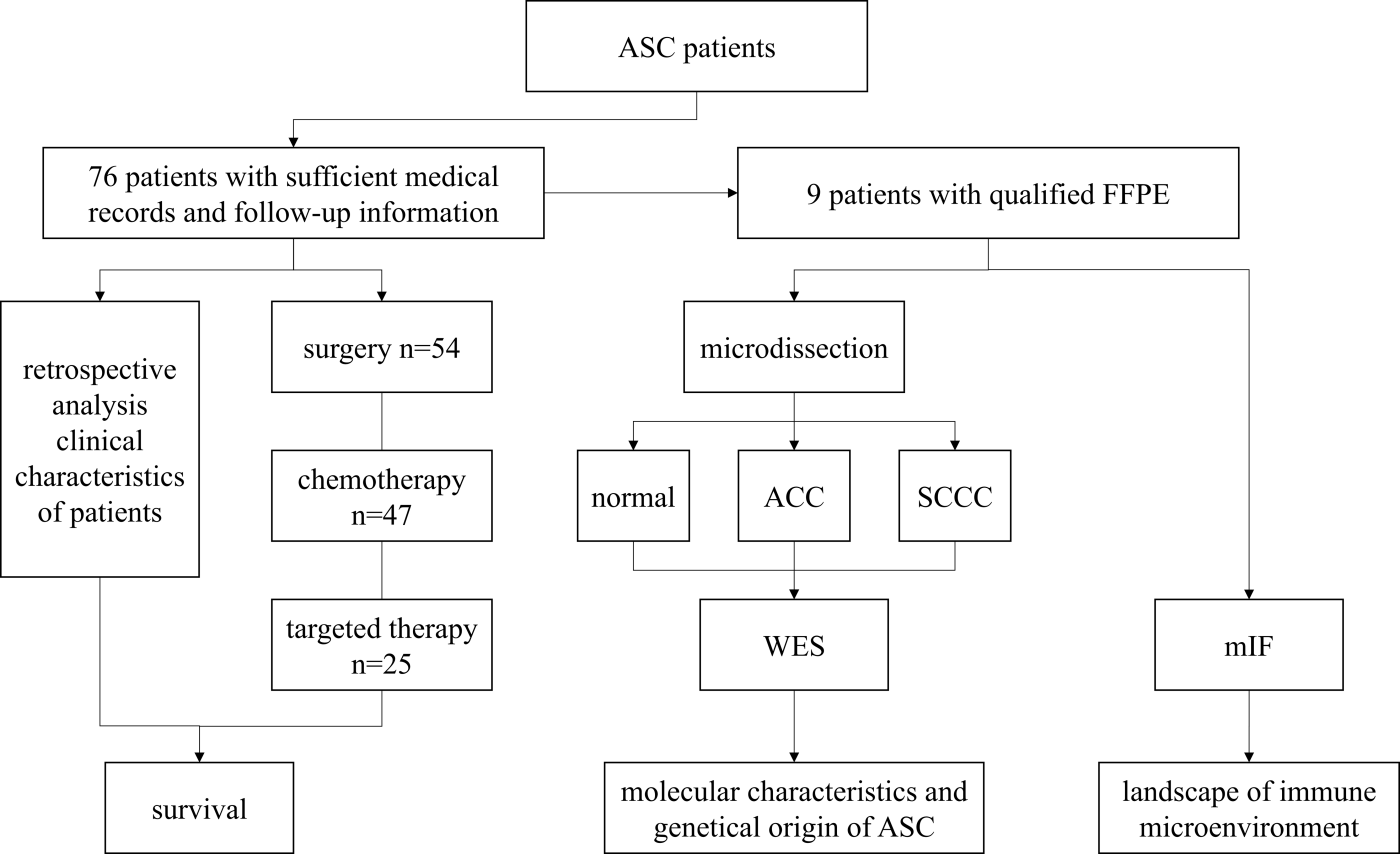
Study workflow. ASC, adenosquamous carcinoma; ACC, adenocarcinoma component; SCCC, squamous cell carcinoma component; WES, whole-exome sequencing; mIF, multiplex immunofluorescence; FFPE, formalin-fixed paraffin-embedded.

The overall study workflow is shown in **Figure 1**. Briefly, 76 patients diagnosed with primary pulmonary ASC were retrospectively enrolled for clinicopathological and survival analyses. Among them, nine patients with sufficient and well-preserved FFPE tumor tissues were selected for microdissection, WES, CNV analysis, phylogenetic reconstruction, and mIF profiling. The clinicopathological characteristics of the 76 patients are summarized in **Table 1**. The cohort included 51 males (67.1%) and 25 females (32.9%), with a median age of 66 years (range: 40-82). Among them, 46 (60.5%) had a history of smoking. According to the 8th edition TNM staging system, the distribution was as follows: stage I (n=25, 32.9%), stage II (n=10, 13.2%), stage III (n=23, 30.3%), and stage IV (n=18, 23.6%). Pleural invasion was observed in 37 cases (48.7%). Treatment modalities included surgical resection in 54 patients (71.1%), chemotherapy in 47 patients (61.8%), radiotherapy in 12 patients (15.8%), targeted therapy in 25 patients (32.9%), and immunotherapy in 18 patients (23.7%). As of the last follow-up, 35 patients were alive and 41 had died, with a median overall survival of 538 days (range: 63-3,992 days). Pathologically, the relative proportions and predominant histological patterns of the ACC and SCCC components were evaluated in all cases. Based on relative ACC and SCCC proportions, a dominant component was defined as one exceeding 60% of the total tumor area. Accordingly, 76 ASC cases were classified into three groups: adenocarcinoma-dominant (n = 33; 43.4%), squamous cell carcinoma-dominant (n = 29; 38.2%), and balanced component (n = 14; 18.4%). ACCs were further subclassified into five histological subtypes: acinar (n = 47; 61.8%), lepidic (n = 10; 13.2%), papillary (n = 9; 11.9%), solid (n = 8; 10.5%), and micropapillary (n = 2; 2.6%). Detailed patient demographic and clinical characteristics are shown (**Table 1**).

**Table 1:** Overview of clinical information of enrolled ASC patients.

Patients (n=76)	
Age	
Median	66
Range	40-82
Gender	
Male	51 (67.1%)
Female	25 (32.9%)
Smoking status	
Ever	46 (60.5%)
Never	30 (39.5%)
Primary site	
LUL	18 (23.7%)
LLL	15 (19.7%)
RUL	21 (27.6%)
RML	4+3 (9.2%)
RLL	15+3 (23.7%)
T stage	
T1	18 (23.7%)
T2	33 (43.4%)
T3	13 (17.1%)
T4	12 (15.8%)
N stage	
N0	38 (50%)
N1-3	38 (50%)
M stage	
M0	58 (76.3%)
M1	18 (23.7%)
Pleural invasion status	
Yes	37 (48.7%)
No	39 (51.3%)
Clinical stage	
I	25 (32.9%)
II	10 (13.2%)
III	23 (30.3%)
IV	18 (23.6%)
Predominant LUAD pattern	
Acinar	47 (61.8%)
Lepidic	10 (13.2%)
Solid	8 (10.5%)
Papillary	9 (11.9%)
Micropapillary	2 (2.6%)
CEA(0.00~5.00ng/mL)	
Median	4.57
Range	0.86-409.22
CYFRA21-1(0.00~3.30ng/mL)	
Median	2.86
Range	0.67-32.68
SCC(0.00~1.50μg/L)	
Median	1
Range	0.3-16
ProGRP(0.00~63.00pg/mL)	
Median	31.33
Range	14.24-81.79
NSE(0.00~16.30μg/L)	
Median	13.475
Range	8.77-30.63
Treatment	
Surgery	54 (71.1%)
Chemotherapy	47 (61.8%)
Radiotherapy	12 (15.8%)
Targeted therapy	25 (32.9%)
Immunotherapy	18 (23.7%)
Survival	
Live	35 (46.1%)
Dead	41 (53.9%)

**Figure 2:**
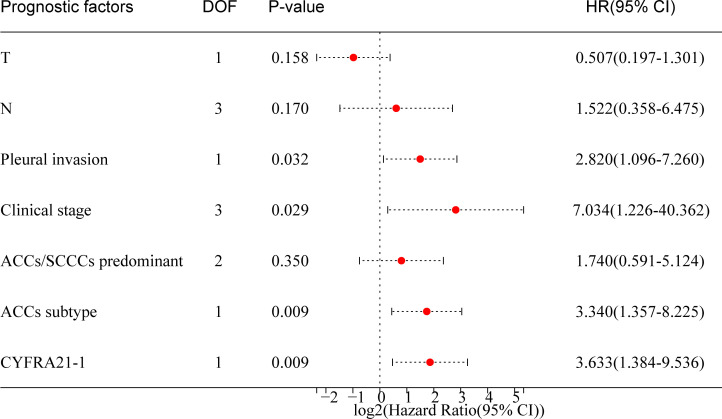
Cox regression analysis showing associations between clinical characteristics and overall survival in ASC patients.

To evaluate the prognostic relevance of these clinical features, univariate and subsequent multivariate Cox regression analyses were performed. As shown in **Figure 2**, four independent prognostic factors were associated with poorer overall survival: the presence of pleural invasion (HR = 2.820, 95% CI 1.096–7.260, P = 0.032), advanced clinical stage (Stage III/IV vs. I/II, HR = 7.034, 95% CI 1.226–40.362, P = 0.029), elevated serum CYFRA21–1 level (HR = 3.633, 95% CI 1.384–9.536, P = 0.009), and the histological subtype of the ACC component. Specifically, patients whose ACC component showed solid or micropapillary patterns had the poorest overall survival (HR = 3.340, 95% CI 1.357–8.225, P = 0.009).

### Clinicopathological characteristics of the nine ASC patients included for WES and mIF analyses

**Figure 3:**
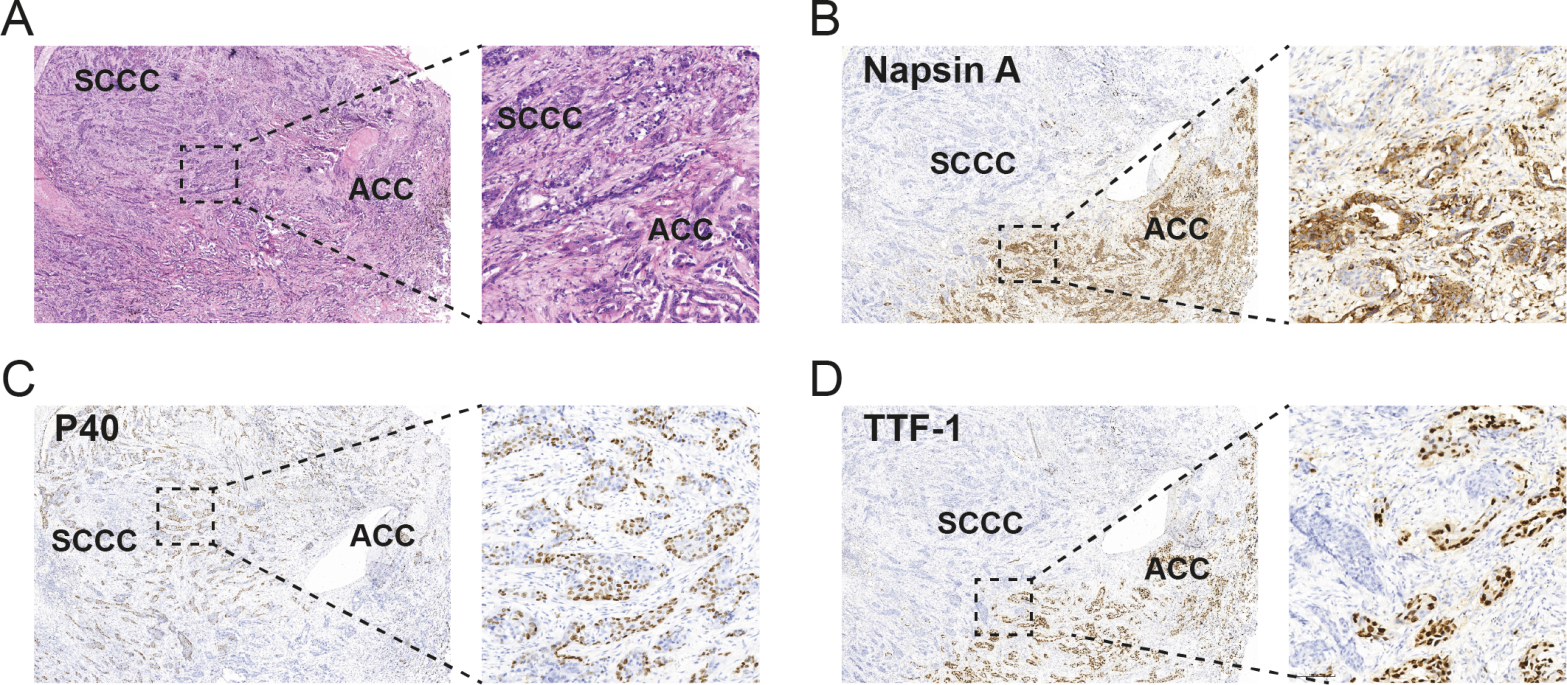
Histopathological and immunohistochemical features of pulmonary adenosquamous carcinoma (ASC). **(A)** A representative hematoxylin & eosinstained ASC section (patient P035), **(B)** ASC immunostained for Napsin A, **(C)** ASC immunostained for P40, **(D)** ASC immunostained for TTF-1.

To reveal the molecular characteristics, clonal evolution patterns and tumor microenvironments corresponding to different ASC components, nine primary tumor samples with sufficient and well-preserved FFPE material were selected for WES and mIF analyses. The main clinicopathological characteristics of these nine patients are summarized (**Table 2**). In terms of clinical staging, one tumor was classified as T3, while the remaining were T1–T2. Two patients had lymph node metastases, one had distant metastasis, and four exhibited pleural invasion. Histopathological examinations confirmed that both ACCs and SCCCs in ASC tissues exhibited morphological features consistent with their respective pure counterparts — conventional adenocarcinoma and squamous cell carcinoma, respectively (**Figure 3A**). A distinct geographical boundary between ACCs and SCCCs was observed by microscopy. Pathologically, all nine ACC samples predominantly exhibited acinar growth patterns, occasionally accompanied by other recognized lung adenocarcinoma subtypes, including lepidic, solid, papillary, and micropapillary patterns. Among SCCCs, features such as keratinization and keratin pearls were observed in some cases. Immunohistochemically, ACC regions stained positively for TTF-1 and Napsin A, while SCCC regions were characterized by positive P40 staining (**Figures 3B–D**).

**Table 2:** Clinico-pathological features of the adenosquamous carcinomas at the time of resection.

Patient	P035	P036	P041	P047	P049	P050	P051	P055	P059
Age(at resection)	66	61	58	50	71	75	64	76	78
Gender	male	male	male	female	female	female	female	male	female
Smoking status	no	no	no	no	no	no	no	yes	yes
TNM classification	pT3N0M0	pT2aN2M1a	pT1bN0M0	pT2aN2M0	pT1cN0M0	pT2aN0M0	pT2aN0M0	pT1bN0M0	pT1bN0M0
Clinical stage	IIb	IVa	Ia2	IIIa	Ia3	Ib	Ib	Ia2	Ia2
Maximum tumor diameter	6.5	4	1.5	2.5	2.5	4	3.5	2	1.4
Lymphatic or venous invasion	no	yes	no	yes	no	no	no	no	no
Pleural invasion	yes	yes	no	yes	no	no	yes	no	no
Spread through air spaces(STAS)	no	no	no	no	no	no	no	no	no
Predominant ACCs pattern	acinar	acinar	acinar	acinar	acinar	acinar	acinar	acinar	acinar
Fraction of ACCs (TTF1+)	40%	≥60%	≥60%	60%	70%	≥60%	20%	55%	50%
Fraction of SCCCs (P40+)	60%	<40%	<40%	40%	30%	<40%	80%	45%	50%

### Genomic characteristics of ACCs and SCCCs in primary ASC

**Figure 4:**
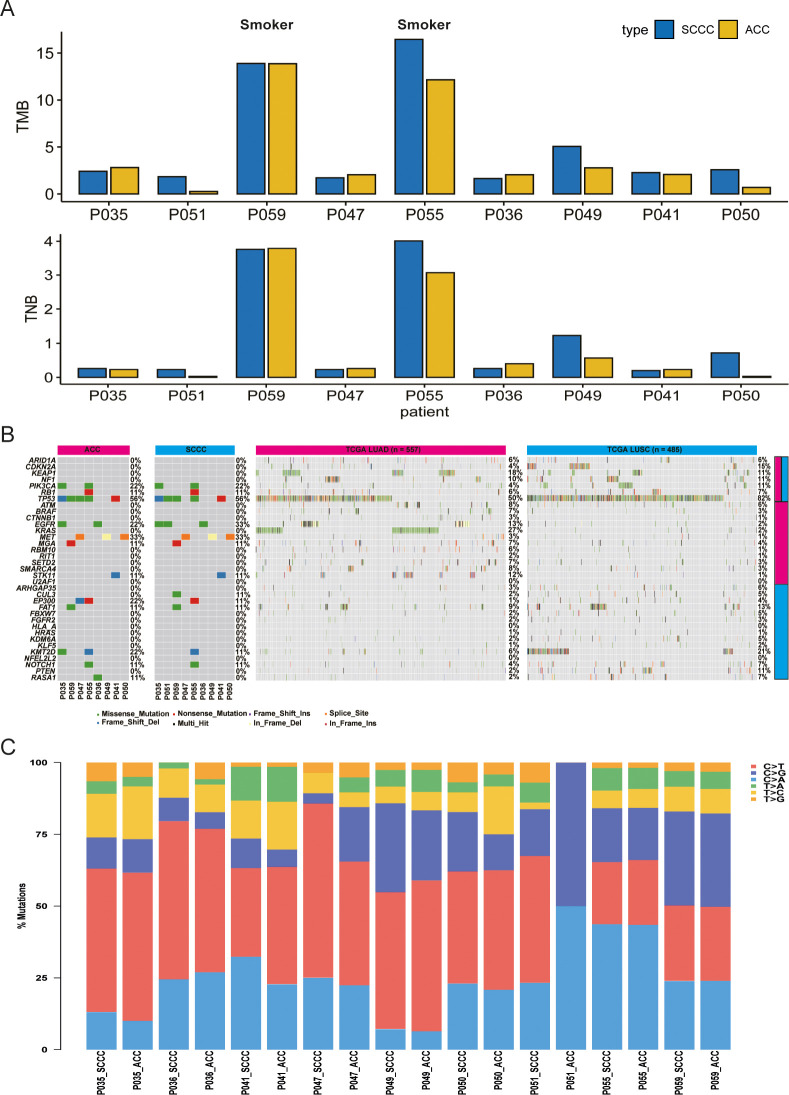
Genomic comparisons between micro-dissected ACCs and SCCCs in ASC tumors. **(A)** Tumor mutational burden (TMB) and tumor neoantigen burden (TNB) in paired ACC and SCCC regions; P055 and P059 were smokers, while others were non-smokers. **(B)** Oncoplot showing non-synonymous mutations based on the gene sets from Bailey et al., comparing across ACC, SCCC, TCGA LUAD, and TCGA LUSC datasets. **(C)** Base substitution pattern distribution in ACCs and SCCCs across patients.

To delineate the genomic landscapes of ACC and SCCC components within pulmonary ASC, we performed microdissection and WES on matched ACC and SCCC regions from nine patients (18 total regions) Across all regions, we identified 2,030 non-synonymous mutations spanning 1,767 genes. The median number of non-synonymous somatic mutations per region was 76. (range: 9 - 552) When including synonymous mutations, the median TMB was 2.34 mutations per megabase (mut/Mb), ranging from 0.26 to 16.45 mut/Mb. Paired analysis revealed that SCCCs regions harbored a significantly higher TMB than their matched ACC counterparts. Tumor neoantigen burden (TNB) followed a similar trend, though the difference did not reach statistical significance. Additionally, a strong association was observed between smoking history and elevated TMB. As shown **Figure 4A**, the two ever-smoker patients (P055 and P059) had significantly elevated TMB compared to never-smokers.

Using MutSigCV, we identified several significantly mutated genes, most frequently TP53 (56%), EGFR (33%), and MET (33%). Comparisons with TCGA data revealed that the TP53 mutation frequency in both ACC (56%) and SCCC (56%) components was intermediate between TCGA LUAD (∼50%) and LUSC (∼82%). Strikingly, EGFR mutation rates in ACC (22%) and SCCC (33%) components were notably higher than in TCGA LUAD (13%) and LUSC (2%) cohorts, respectively. Similarly, MET mutation rate (33%) was significantly enriched in our cohort versus both TCGA LUAD and LUSC. No BRAF or KRAS mutations were detected (**Figure 4B**). We further examined the co-occurrence of major driver mutations. Within this limited WES cohort, EGFR and MET mutations were each detected in 3 of 9 cases, whereas KRAS mutations were absent. EGFR- and MET-mutated tumors occurred in different cases, and no co-occurrence of EGFR, KRAS, and MET driver mutations was observed.

Analysis of base substitution patterns showed that C>T and C>A substitutions were predominant, together accounting for more than 50% of all somatic mutations (**Figure 4C**). Matched ACC and SCCC regions within the same tumor exhibited highly similar substitution profiles, supporting a shared mutational background. Notably, the smoker case P055 showed a markedly increased frequency of C>A transversions compared with non-smokers, consistent with a tobacco-related mutational signature and previous reports linking smoking exposure to elevated C>A transversions in lung cancer (24, 25).

### Somatic CNVs in human ASC tumors

**Figure 5:**
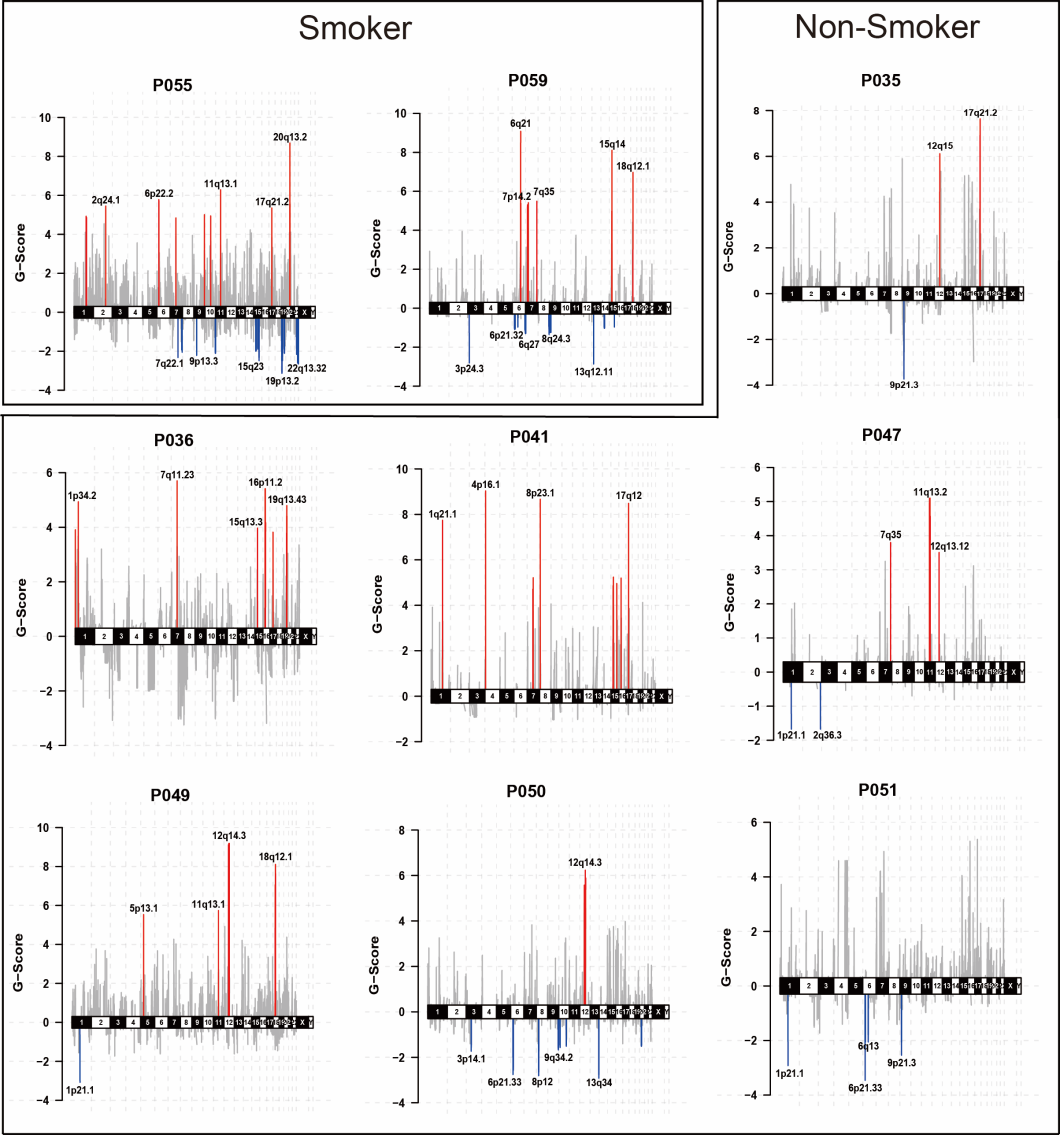
GISTIC2.0-based G-score analysis of CNVs across ASC patients. G-score above 0 indicate amplification (red); G-score below 0 indicate deletion (blue).

CNV analysis of tumor components from nine ASC patients revealed high concordance between ACC and SCCC regions in the magnitude (log_2_ ratios), number, and chromosomal locations of copy number variations (**Figure 5**; **Supplementary Figure 1**). Global somatic CNV analysis using GISTIC2.0 (G-SCORE) identified several genomic regions with significant amplifications or deletions. For instance, P036 and P041 cases predominantly exhibited amplification events, while P051 showed only deletions. Although amplifications and deletions appeared randomly across chromosomes, certain loci exhibited recurrent alterations; notably, recurrent amplification regions included 11q13.1, 17q21.2, and 7q35, while deletions frequently occurred in 1p21.1, 6p21.3, and 9p21.3 regions.

It is well established that tobacco smoke exposure significantly increase mutation rates in lung cancers (22, 26). To further investigate the influence of environmental exposure, CNVs were stratified by smoking status. In line with this, smoking-associated ASC cases (P055 and P059) exhibited a substantially higher CNV burden than non-smokers. Specifically, amplification regions in P055 were enriched in 2q24.1, 6p22.2, 11q13.1, 17q21.2, and 20q13.2 regions, while P059 showed amplification peaks in 6q21, 7p14.2, 7q35, 15q14, and 18q12.1 regions. Deletion regions in P055 were mainly located at 7q22.1, 9p13.3, 15q23, 19p13.2, and 22q13.32 regions, whereas P059 exhibited deletions in 3p24.3, 6p21.32, 6q27, 8q24.3, and 13q12.11 regions. Across both smoking and non-smoking patients, deletion events were generally fewer and of lower amplitude than amplifications. This pattern possibly reflected a greater likelihood of clonal expansion and the selective retention of amplification regions during tumor evolution.

### Shared clonal origin of ACC and SCCC components

**Figure 6:**
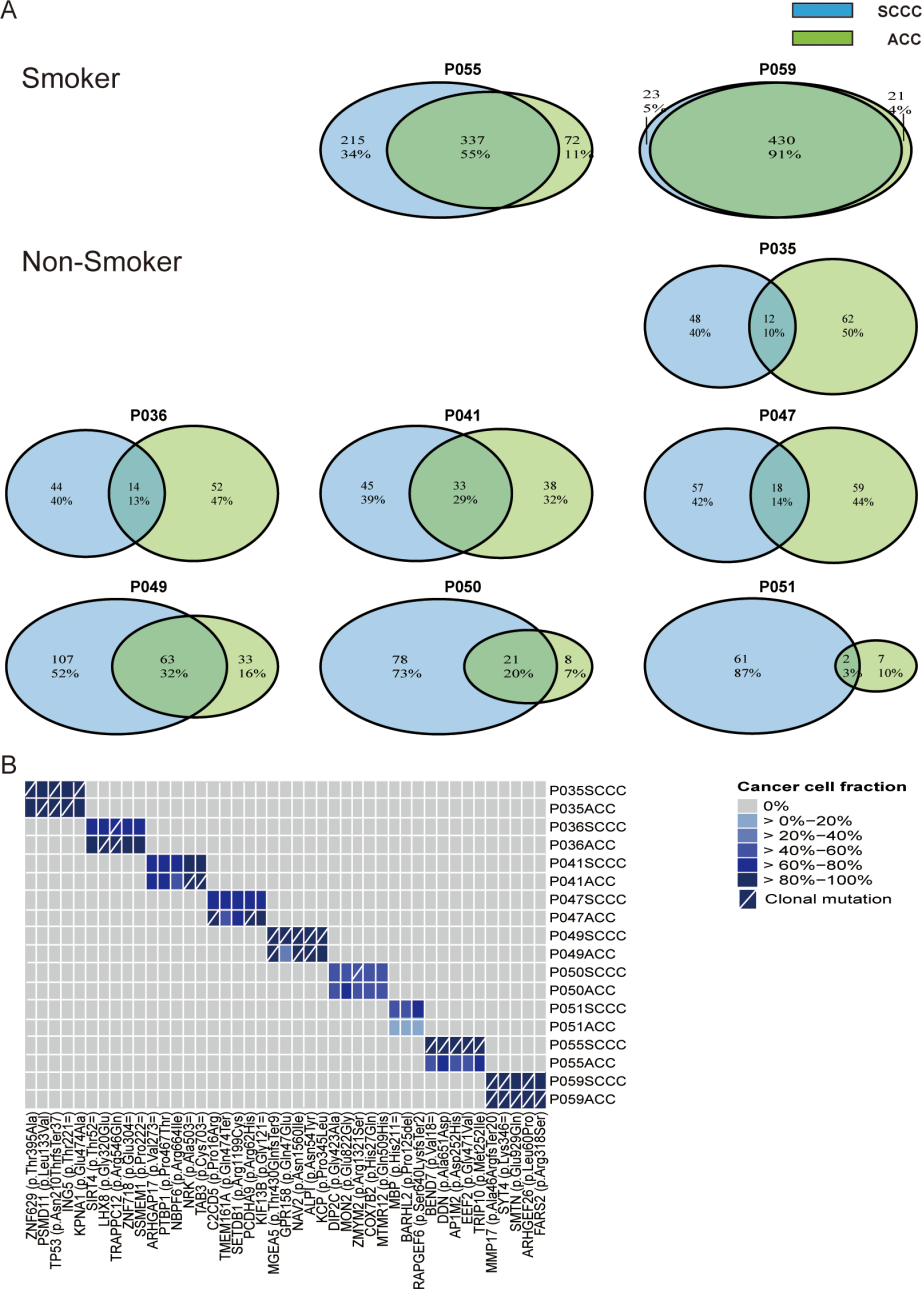
Evidence for the monoclonal origin of ACCs and SCCCs in ASC tumors. **(A)** Venn diagrams show the number of shared non-synonymous mutations between paired ACC (green) and SCCC (blue) regions. Circle size is proportional to the mutation count. **(B)** Heatmap showing the cancer cell fraction (CCF) for selected mutations. Diagonal lines indicate clonal mutations.

To investigate clonal origin in ASC tumors, we analyzed the distribution of shared and private mutations between ACCs and SCCCs in individual patients. Venn diagrams illustrated that all nine patients exhibited a subset of shared non-synonymous mutations between matched ACC and SCCC regions, with the proportion of shared non-synonymous mutations in the 3–91% range (**Figure 6A**). Notably, in two smoking-associated cases (P055 and P059), the proportion of shared mutations was substantially higher (P055: 55%; P059: 91%) compared to non-smokers, who exhibited only 3–32% shared mutations.

To further assess clonal architecture, we calculated the CCF of mutation sites. For each case, the five mutations with the highest VAF were selected and plotted, with clonal mutations labeled by diagonal hatching. Our analysis revealed that ACCs and SCCCs within the same tumor not only shared multiple somatic mutations but also exhibited both clonal and subclonal features (**Figure 6B**). These observations supported the hypothesis that the two histologically distinct components (ACCs and SCCCs) originated from a common ancestral clone, consistent with a monoclonal ASC origin.

### Distinct evolutionary trajectories in smoking and non-smoking ASC

**Figure 7:**
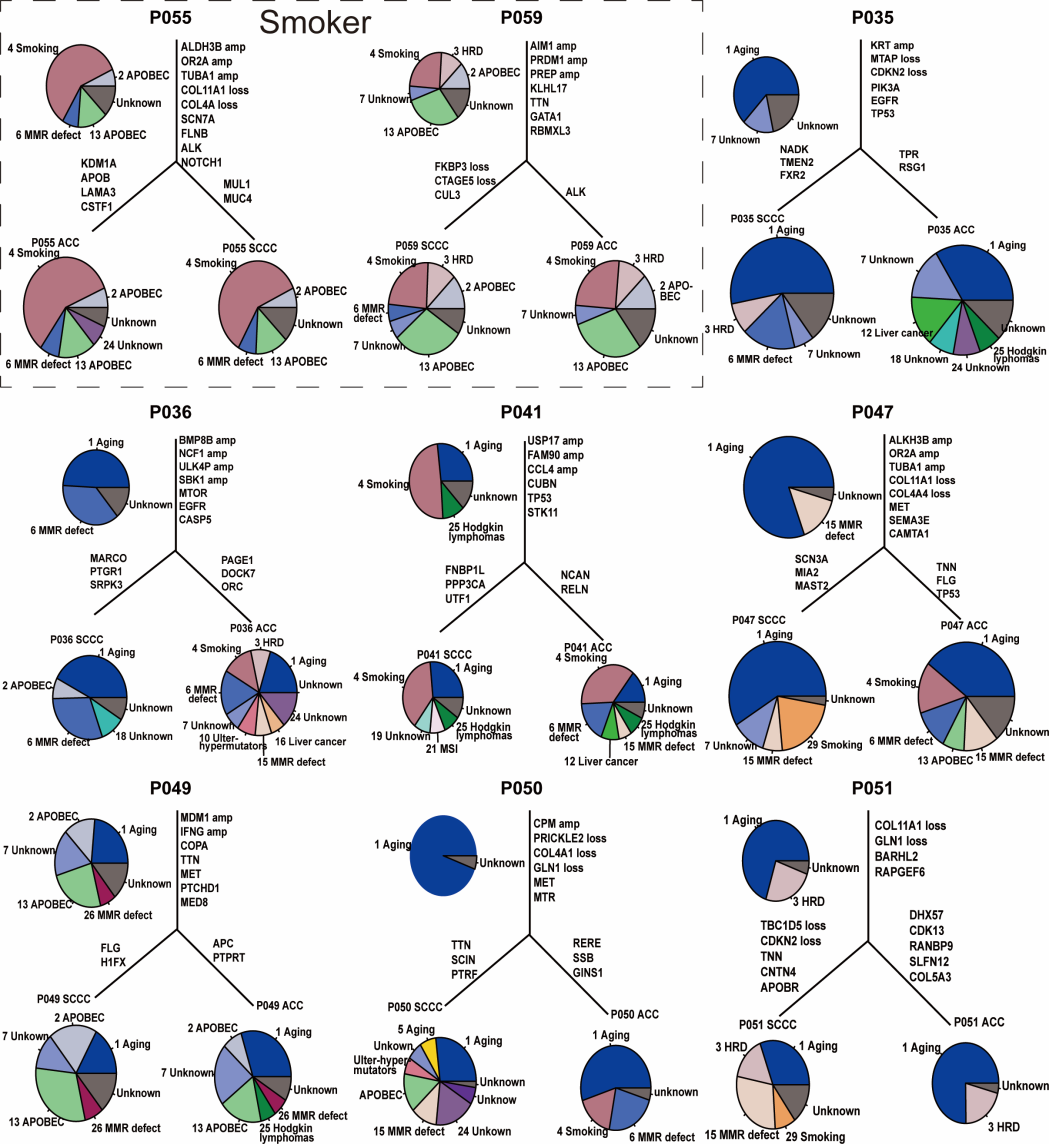
Evolutionary trajectory and mutational signatures in ASC tumors. Maximum parsimony trees show trunk and branch mutations in ACCs and SCCCs. Pie charts represent mutational signature proportions. Vertical lines denote trunk mutations; diagonal lines denote ACC or SCCC branches. Cancer gene alterations are annotated. P055 and P059 were smokers and other patients were non-smokers.

To investigate evolutionary trajectories in ASC tumors, maximum parsimony phylogenetic trees were constructed for each case. Phylogenetic analysis revealed that the majority of somatic mutations were located on trunks rather than branches (**Figure 7**), indicating that these early events likely represented clonal and potentially driver alterations. A total of 96 key genomic alterations — including point mutations and CNVs—were mapped across trunks and branches in evolutionary trees. Among these altered genes, many were known lung cancer drivers, including APC, MET, STK11, and CUL3, the latter of which is commonly implicated in squamous cell carcinoma. Shared driver mutations across both histological types included EGFR, NOTCH1, TP53, and ALK. Specific trunk mutation examples included EGFR in P035 and P036; MET in P047, P049, and P050; and TP53 in P035 and P041. Interestingly, TP53 also appeared on the adenocarcinoma branch in P047. ALK mutations were found on the trunk in P055 and on the adenocarcinoma branch in P059. These findings suggested that key oncogenic mutations arose early and were retained through subsequent evolutionary divergence.

Mutational signature analysis was performed using the 30 classic COSMIC signatures (v.2). In two smoker-associated cases (P055 and P059), both trunk and branch mutations were dominated by mutational signature 4 (smoking-associated) and APOBEC-associated signatures. In contrast, non-smoker tumors predominantly exhibited an aging-related signature 1 in their trunk mutations, with consistent Aging signatures also observed in both ACC and SCCC branches. An exception was patient P041, whose trunk displayed both Smoking and Aging signatures, suggesting possible exposure to second-hand smoke. Similarly, evidence of passive smoking was inferred in branch mutations of non-smoking patients P036, P050, and P051, which also exhibited smoking-related features. Interestingly, in patient P047 — who had no smoking history — signature 29 (associated with chewing tobacco) was identified in the SCCC branch, while signature 4 (Smoking) was detected in the ACC branch. This suggested possible exposure to smokeless tobacco products. Notably, the extent of APOBEC-related mutagenesis was greater in P059 than in P055, potentially reflecting differences in their smoking histories or exposure intensity. Another key distinction between smoking and non-smoking groups was evident from their evolutionary branch complexity. Tumors from smokers (e.g., P055 and P059) displayed phylogenies with structurally simpler, less divergent branches. In contrast, tumors from non-smoking exhibited greater branching complexity and a wider diversity of mutational processes operative in the subclonal (branch) compartments (**Figure 7**). These findings underscored distinct evolutionary trajectories driven by smoking versus non-smoking etiologies in ASC.

### Enhanced immune cell infiltration in ACCs compared to SCCCs in ASC tumors

**Figure 8:**
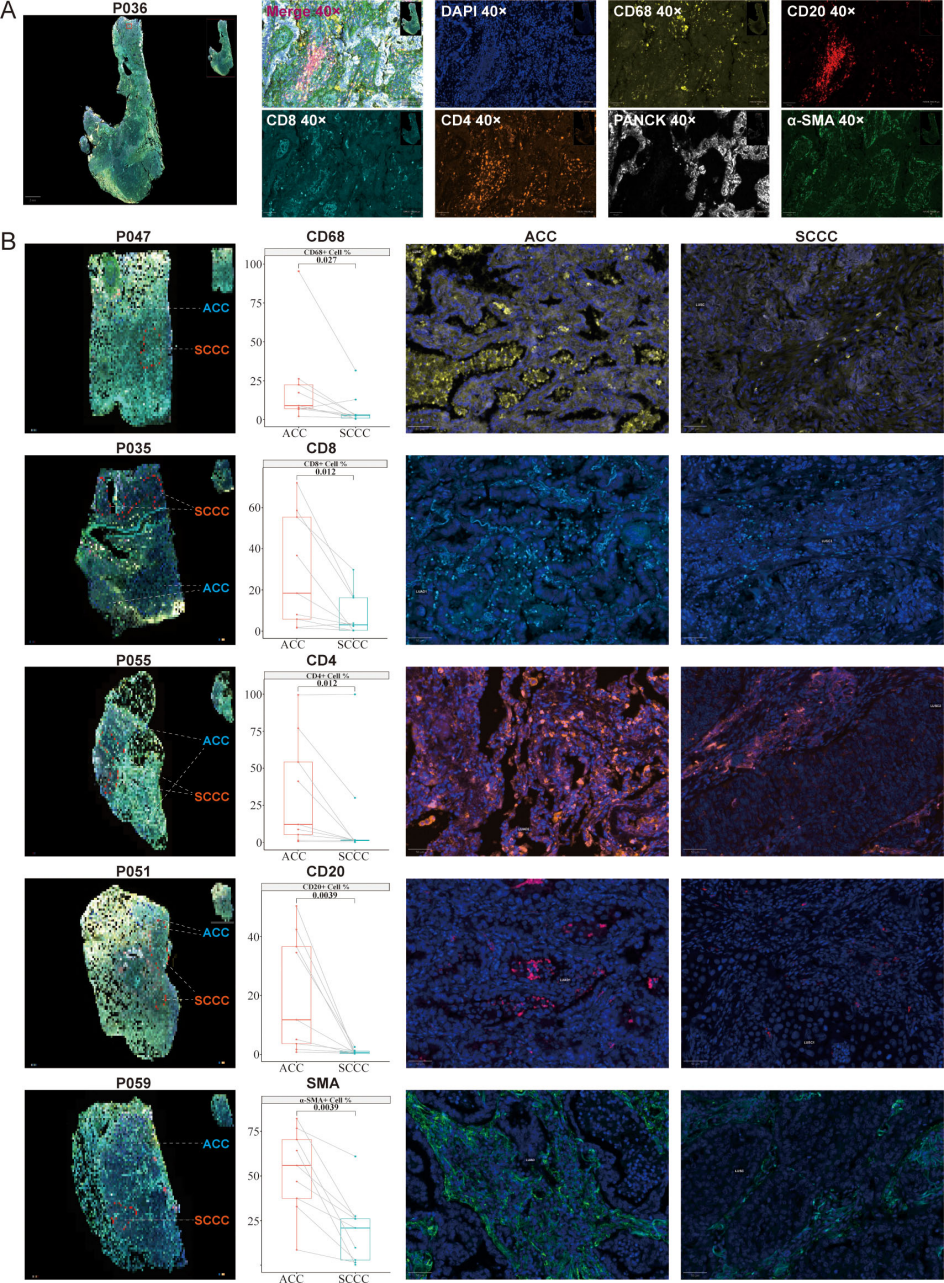
Representative multiplex immunofluorescence staining in ASC tissues. **(A)** Whole-slide merged image and 40× magnification showing individual markers: DAPI, CD68, CD20, CD8, CD4, PanCK, and α-SMA. **(B)**. Quantitative comparisons and representative imaging of immune cell and cancer-associated fibroblast infiltration in paired ACC and SCCC regions in ASC tumors.

Using mIF, we compared immune cell and CAF infiltration between paired ACC and SCCC regions from nine ASC cases. ACC regions exhibited significantly higher CD8^+^ T cell, CD4^+^ T cell, CD20^+^ B cell, CD68^+^ macrophage, and α-SMA^+^ CAF infiltration than paired SCCC regions, in terms of both cell density and relative proportion (**Figure 8**). Spatial analysis showed no significant differences between ACC and SCCC regions, except that CD4^+^ T cells were located farther from Pan-CK-labeled tumor cells in ACC regions (**Supplementary Figure 2**). These results suggest greater immune infiltration in ACC regions.

## Discussion

Although pulmonary ASC is a rare histological subtype among NSCLC, its unique pathological features, molecular complexity, and poor clinical prognosis outcomes underscore its significant research value. Currently, the management of ASC largely follows general NSCLC treatment guidelines, lacking subtype-specific therapeutic strategies. The main reason lies in the insufficient understanding of its clonal origin, molecular driving mechanisms, and TME heterogeneity. In this study, we systematically characterized the clinical-genomic-immunological landscape of ASC by integrating survival analysis, WES, and mIF profiling, thereby providing a key basis for the development of precision treatment strategies.

In a cohort of 76 patients with ASC, we identified visceral pleural invasion (VPI), advanced clinical stage (III/IV), elevated serum CYFRA21–1 levels, and specific pathological subtypes of ACC (solid type and micropapillary type) as independent prognostic risk factors. This result not only aligns with the general prognostic pattern of NSCLC but also highlights the unique biological characteristics of ASC. Visceral pleural invasion is a well-recognized marker of aggressive tumor behavior in lung cancer and is notably prevalent in ASC (27–29). In a previously reported cohort of 59 ASC patients reported by Guo et al., VPI was observed in 41 cases (69.5%) and was validated as an independent prognostic factor, with a median overall survival of 13.6 months and 1- 3- and 5-year survival rates of 59.9%, 36.4%, 31.2%, respectively (30). In our larger cohort of 76 ASC patients, VPI was present in 37 cases (48.7%), and multivariate analysis confirmed its significant association with poorer survival outcomes, consistent with prior reports. Additionally, this study systematically revealed the stratification value of ACC pathological subtypes for the survival of ASC patients, suggesting that the solid type and micropapillary type ACC have higher invasiveness, and the mechanism may involve decreased intercellular adhesion ability, enhanced interstitial infiltration, and the formation of an immunosuppressive tumor microenvironment. This finding is consistent with the adverse prognostic characteristics of similar subtypes in lung adenocarcinoma. Serum CYFRA21-1, a biomarker derived from squamous epithelial cells, correlates positively with the proliferative activity of the SCCC, offering a practical and non-invasive tool for assessing tumor aggressiveness. The integration of multiple prognostic variables enables the construction of a more refined risk stratification model for ASC.

Firstly, WES analysis revealed that ASC harbors a unique mutational profile distinct from both LUAD and LUSC, and partially integrates the molecular characteristics of both, presenting significant subtype-specificity. In this cohort, TP53 (56%), EGFR (33%), and MET (33%) were frequently mutated genes. Among them, the mutation rate of EGFR in ACC (22%) and SCCC (33%) was significantly higher than the reported values in LUAD (13%) and LUSC (2%) in TCGA database; the mutation rate of MET (33%) was also at a relatively high level in both ACC and SCCC components. Importantly, canonical driver mutations commonly found in LUAD, such as BRAF and KRAS, were absent in this cohort. These observations suggest that ASC may follow a distinct molecular pathogenesis. TP53, a core tumor suppressor gene, is frequently altered in ASC; its mutations disrupt G1/S and G2/M cell cycle checkpoints, leading to genomic instability and serving as a “foundational event” in tumorigenesis—a feature highly concordant with the early driver role of TP53 mutations (~82%) in LUSC. Concurrently, activating mutations in EGFR and MET can constitutively activate downstream PI3K-AKT and RAS-MAPK signaling pathways, promoting tumor cell proliferation, survival, invasion, and metastasis. The high prevalence of these alterations identifies them as promising therapeutic targets for precision oncology in ASC.

Clonal analysis and phylogenetic reconstruction revealed a monoclonal origin for ASC: the ACC and SCCC components share a substantial number of core driver mutations (e.g., TP53, EGFR), with smoking patients exhibiting a significantly higher proportion of shared mutations (55%–91%) compared to non-smokers (3%–32%). Moreover, both components display high concordance in copy number variation (CNV) profiles, including recurrent 11q13.1 amplification and 9p21.3 deletion. These findings support a model in which ASC arises from a common progenitor cell and diverges into two histological phenotypes through branched evolution—a hypothesis consistent with the “clonal differentiation” model proposed by Krause et al. based on WES data.

Smoking status emerges as a pivotal determinant of ASC’s molecular evolutionary trajectory. In smokers, tumor evolution follows a “simple branching” pattern, with trunk and branch mutations predominantly shaped by tobacco-related mutational signatures (COSMIC signature 4) and APOBEC-associated processes. In contrast, non-smokers exhibit mutational profiles dominated by age-related signatures (COSMIC signature 1) and more complex phylogenetic architectures, indicative of increased subclonal diversification. Additionally, smokers exhibit significantly higher tumor mutation burden (TMB) and CNV burden than non-smokers, mirroring the smoking-associated hypermutated phenotype observed in LUSC. These results elucidate how environmental exposures shape genomic evolution and provide a theoretical rationale for immune checkpoint inhibitor therapy in smoking-related ASC—high TMB is typically associated with increased neoantigen load, enhancing tumor immunogenicity and improving the likelihood of response to immunotherapy.

The immune characteristics of the TME are key determinants of immunotherapy efficacy. Previous studies have generally shown that LUSC tends to exhibit higher PD-L1 expression and greater immune infiltration than LUAD, although marked intertumoral heterogeneity exists in both subtypes (13). In the present study, paired analysis within ASC tumors showed that ACC regions exhibited significantly higher infiltration of CD8^+^ T cells, CD4^+^ T cells, CD20^+^ B cells, CD68^+^ macrophages, and α-SMA^+^ CAFs than matched SCCC regions. This finding suggests that the immune microenvironment of ASC does not simply recapitulate the typical immune features of either LUAD or LUSC but instead displays component-specific immune heterogeneity. In recent years, immune checkpoint inhibitors have become an important therapeutic strategy for NSCLC, and their potential role in pulmonary ASC has also begun to attract attention. Although prospective ASC-specific clinical trials are still lacking, retrospective studies and case reports suggest that PD-1/PD-L1 blockade, either alone or combined with chemotherapy, may provide clinical benefit in selected patients with advanced ASC (31). In our cohort, the relatively abundant immune infiltration observed in ACC regions and the higher TMB in smoking-associated ASC cases may suggest potential sensitivity to immune checkpoint blockade in a subset of patients. However, because our study lacked systematic PD-L1 assessment, clinical immunotherapy response data, and independent LUAD or LUSC control cohorts, we cannot determine whether ASC tumors are globally more immune-rich or more likely to respond to immunotherapy than conventional LUAD or LUSC. Future studies with larger cohorts integrating PD-L1 expression, TMB, spatial immune profiling, and treatment-response data are needed to clarify the immunotherapeutic relevance of ASC immune heterogeneity.

One limitation of this retrospective study is that standardized differentiation grading for the ACC and SCCC components was not consistently available in the original pathological records. Therefore, we could not reliably assess the prognostic impact of differentiation status for each histological component. Future studies with standardized pathological evaluation are needed to clarify the prognostic relevance of differentiation status in pulmonary ASC. In addition, although our mIF analysis revealed increased CD8^+^ and CD4^+^ T-cell infiltration in ACC regions, the current antibody panel was not designed to evaluate the functional status of these T cells. Therefore, we could not determine whether the infiltrating T cells were predominantly activated or exhausted. Future studies using spatial transcriptomics and/or expanded mIF panels incorporating activation markers such as GZMB, IFN-γ, Ki-67, CD69, and HLA-DR, as well as exhaustion markers such as PD-1, TIM-3, LAG-3, TIGIT, and TOX, are warranted to better characterize T-cell functional states and their spatial relationships with ASC tumor components.

## Conclusions

This study provides a comprehensive genomic and immune profiling of ASC, revealing that ACC and SCCC components shared a common clonal origin despite their distinct histological features. The molecular genetic features and tumor microenvironment in ASC displayed marked heterogeneity between ACC and SCCC regions within ASC. Smoking status is the core factor driving the molecular evolution trajectory of ASC. These findings elucidate the unique molecular profile and potential therapeutic targets of ASC and provide a critical foundation for developing component-based personalized treatment strategies.

## Data Availability

The original contributions presented in the study are included in the article/**Supplementary Material**, further inquiries can be directed to the corresponding author/s.

## Supplementary material

Supplementary Figure 1 Pairwise CNV concordance in ASC Tumors: scatter plots of CNV in matched ACC vs SCCC regions from nine patients.

Supplementary Figure 2 Spatial proximity analysis between tumor cells and microenvironmental subpopulations in paired ACC and SCCC regions.
