# Supplementary figures and images for: Integrated genomic and immunophenotypic profiling reveals monoclonal origin, smoking-driven evolution and heterogeneous microenvironment in pulmonary adenosquamous carcinoma
Source: Front Immunol. 2026 Jun 15;17:1865105. doi: 10.3389/fimmu.2026.1865105 (PMC13311056; doi:10.3389/fimmu.2026.1865105)

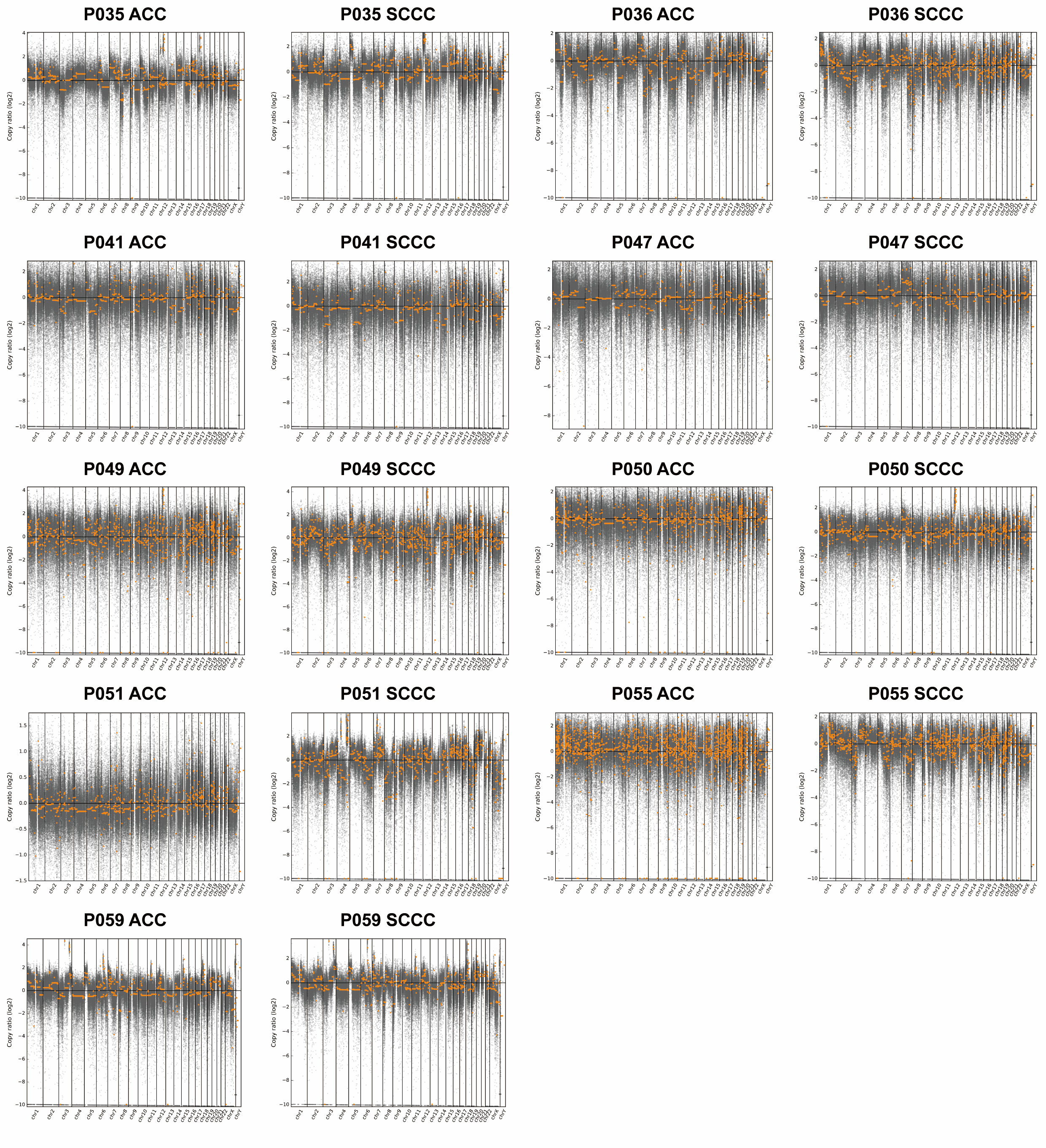

Supplement: Supplementary Figure 1 — Pairwise CNV concordance in ASC Tumors: scatter plots of CNV in matched ACC vs SCCC regions from nine patients. [file Image1.tif]

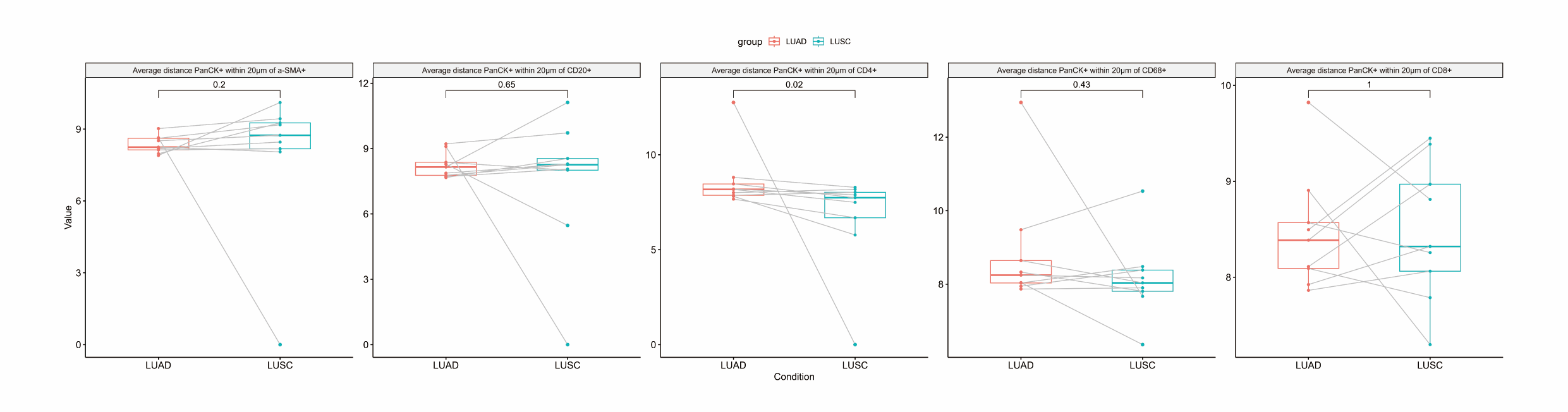

Supplement: Supplementary Figure 2 — Spatial proximity analysis between tumor cells and microenvironmental subpopulations in paired ACC and SCCC regions. [file Image2.tif]
